# pyAIR—A New Software Tool for Breathomics Applications—Searching for Markers in TD-GC-HRMS Analysis

**DOI:** 10.3390/molecules27072063

**Published:** 2022-03-23

**Authors:** Lilach Yishai Aviram, Dana Marder, Hagit Prihed, Konstantin Tartakovsky, Daniel Shem-Tov, Regina Sinelnikov, Shai Dagan, Nitzan Tzanani

**Affiliations:** 1 Department of Analytical Chemistry, Israel Institute for Biological Research1, P.O. Box 19, Ness Ziona 7410001, Israel; lilachy@iibr.gov.il (L.Y.A.); danam@iibr.gov.il (D.M.); hagitp@iibr.gov.il (H.P.); shaid@iibr.gov.il (S.D.); 2 Scent Medical Technologies, Rehovot 7670107, Israel; konstantin@scentech-medical.com (K.T.); dstov101@gmail.com (D.S.-T.); regina@scentech-medical.com (R.S.)

**Keywords:** breath analysis, VOCs, pyAIR, MS-DIAL, GC-MS (Orbitrap), TD-GC-HRMS

## Abstract

Volatile metabolites in exhaled air have promising potential as diagnostic biomarkers. However, the combination of low mass, similar chemical composition, and low concentrations introduces the challenge of sorting the data to identify markers of value. In this paper, we report the development of pyAIR, a software tool for searching for volatile organic compounds (VOCs) markers in multi-group datasets, tailored for Thermal-Desorption Gas-Chromatography High Resolution Mass-Spectrometry (TD-GC-HRMS) output. pyAIR aligns the compounds between samples by spectral similarity coupled with retention times (RT), and statistically compares the groups for compounds that differ by intensity. This workflow was successfully tested and evaluated on gaseous samples spiked with 27 model VOCs at six concentrations, divided into three groups, down to 0.3 nL/L. All analytes were correctly detected and aligned. More than 80% were found to be significant markers with a *p*-value < 0.05; several were classified as possibly significant markers (*p*-value < 0.1), while a few were removed due to background level. In all group comparisons, low rates of false markers were found. These results showed the potential of pyAIR in the field of trace-level breathomics, with the capability to differentially examine several groups, such as stages of illness.

## 1. Introduction

The human respiratory system emits a variety of volatile organic compounds (VOCs). The major portion of high-concentration VOCs in breath are from the surrounding environment and are not related to metabolic activity. The minority are products of human metabolism. These products originate from two sources: the respiratory system itself and by diffusion from blood—systemic compounds that pass the blood–air barrier in the lungs [1,2]. These metabolism-originating compounds are usually present at low concentrations in the nL/L range and are even lower. Breathomics, as a branch of metabolomics, is aimed at the targeted and non-targeted analysis of volatile compounds in exhaled breath, in the pursuit after insights on physiological state.

Since the process of collecting VOCs exhaled from the lungs (exhaled air) is non-invasive and straightforward, breath analysis has the potential as a valuable clinical diagnostic tool. VOCs in exhaled breath have been correlated with the pathophysiology of cancers and other pulmonary diseases, as well as gastric diseases [3,4,5,6,7]. It was found that in many cases, VOC biomarkers were not exclusive to sick individuals but differed in their concentration compared to individuals that were healthy or had other illnesses [8,9]. For instance, heptane in a healthy population is usually around 0.3 ng/mL, whereas in sick individuals with chronic obstructive pulmonary disease (COPD), it was found to be around 1 ng/mL. On the other hand, some reports claim to have found VOCs that are unique to a specific disease [10]. 

The analysis of volatile metabolites Is generally carried out by gas chromatography mass spectrometry (GC-MS), operated with electron ionization (EI) [11,12]. GC-MS is a highly selective technique that can separate, detect, identify, and quantify VOCs at low- to sub- ng/mL levels [13]. The peaks obtained by GC-MS can be identified by matching measured spectra with spectra in a reference library, which takes into consideration not only the mass and intensities of the ions obtained but also the chromatographic retention index (RI) of the compound of interest [14]. Hence, the use of GC-MS for targeted and non-targeted metabolic studies, especially for breathomics, is highly suitable and powerful [15]. In recent years, GC-HRMS has gained popularity [16]. GC-Orbitrap provides a high mass resolving power (120,000 full width at half maximum (FWHM) at (*m*/*z* 200)) combined with a high mass accuracy (<5 ppm), which is needed to avoid isobaric interference [17]. The capabilities of GC-Orbitrap have already been demonstrated in targeted breath biopsies [18] and untargeted metabolomics in blood [19]. While high-resolution data from Orbitrap GC-MS only slightly improve library matching compared to conventional GC-MS, it better supports the correct structure elucidation of an unknown in metabolomics studies [20,21]. The vendor-designated data processing software tool for untargeted metabolomics on GC-Orbitrap is the “Compound-Discovere”, which handles all the required steps in data analysis, including peak detection, data deconvolution, peak area integration, background filtering, and library identification [22]. An alternative approach is to implement a platform-independent software tool compatible with GC-HRMS data. Among the prominent packages with a higher level of documentation and support, we find tools such as XCMS (web-based or user installed) [23], mZmine 2 [24], metaMS [25], MetAlign [26], El-MAVEN [27], and MS-DIAL [28]. These tools process the raw data (usually after conversion to a common file format), detect the compounds in the sample, and compare the different groups in order to highlight the distinguished ones. Next, the putative markers are identified using MS-spectral libraries, such as NIST, MZcloud, or others [29], and investigated online in metabolism-related databases [30,31] or general chemical-entity databases (Pubchem, Chemspider) for more information about their biological context. 

The application of GC-MS in the field of untargeted metabolomics is challenging because EI energetic fragmentation coupled with single-stage MS hampers peak detection and deconvolution. This challenge is more pronounced at low concentrations of analytes in the presence of matrix interference. The high resolution and high mass accuracy of GC-Orbitrap improves the capability to differentiate between isobaric ions and calculate elemental composition. However, the low concentrations of the metabolites of interest result in low S/N ratios and induce variability in the extracted spectra. Breathomics research brings in an additional complexity that stems from the relatively low mass of the compounds of interest (with their EI fragments) having a high similarity in molecular composition. Therefore, the correct alignment of compounds between samples and between groups of samples becomes a key step in the data processing workflow before statistical analysis of the results can take place.

In this paper, we report an in-house development of a new software tool called pyAIR, suitable for the detection of biomarkers-small VOCs, in breath analysis. pyAIR is part of a complete workflow for the detection of untargeted biomarkers in air samples using TD-GC-Orbitrap. The software uses accurate mass data, performs alignment of compounds across samples and groups (using RT and spectra similarity), and uses statistical tools to look for markers in each group. pyAIR can handle complex data matrices of hundreds of samples while representing variability, such as different stages of illness. Our workflow was tested in the differential analysis of four groups of samples spiked with small VOCs down to sub-nL/L level, achieving successful untargeted detection of all markers.

## 2. Results

To evaluate the performance of the proposed workflow for detecting discriminating compounds, we carried out an experimental setup that simulates the analysis of several groups of breath samples. 

### 2.1. Gas Samples and Model Analytes

For this simulation, 27 small VOCs were selected as model analytes to simulate possible markers in breathomics. These VOCs were spiked as a mixture at different concentrations into Tedlar bags filled with pure nitrogen. From each bag, 0.3 L of gas was pumped into a TD tube to prepare the samples for analysis using TD-GC-HRMS. These spiked samples were used as a controlled probe to examine the performance of pyAIR, simulating possible scenarios in untargeted breathomics. The 27 VOCs represent a variety of functional groups—alkanes, alcohols, ethers, ketones, and esters—with a molecular mass in the range of 60–136 Da, as detailed in Table 1. A mixture of the metabolites was used to prepare a high concentration Tedlar bag sample, with a uniform concentration of 200 μL/L for all compounds. This stock sample was consecutively diluted to 13 samples at seven concentrations in Tedlar bags (as described in the experimental part): Nitrogen-Blank, 2 × 0.3 nL/L, 2 × 1 nL/L, 2 × 3 nL/L, 2 × 10 nL/L, 2 × 30 nL/L and 2 × 100 nL/L, two Tedlar bags for each concentration. From each bag, 0.3 L of gas was pumped into a TD tube to prepare the samples for analysis.

After analysis of the tube samples using TD-GC-MS (Orbitrap), the compounds (“markers”) were identified and plotted on calibration curves. A high linear fit was found for 24 out of the 27 compounds (R^2^ > 0.99) in the range of 0.3–30 nL/L. Propanol and 2-methoxy ethanol were not detected at the lower concentration (0.3 nL/L), hence their linear range was 1–30 nL/L. Phenol was detected at high concentrations in all samples as part of the background. While processing the analysis results, the samples were divided into three groups, simulating different stages of “illness”: blank (1 × pure nitrogen), group L (Low: 2 × 0.3 nL/L and 2 × 1 nL/L), group M (Medium: 2 × 3 nL/L and 2 × 10 nL/L), and group H (High: 2 × 30 nL/L and 2 × 100 nL/L). The separation into groups simulates varying VOC concentrations in exhaled breath (3.3-fold change within each group), with a 10-fold difference in the averaged concentration of the markers between adjacent groups. There is a 3-fold difference in the concentration of the markers between the lower concentration of H and M groups and the higher concentration of M and L groups, respectively. This experimental design enabled us to test and evaluate the performance of both the analytical system (TD-GC-MS) and pyAIR as part of the data processing pipeline in terms of sensitivity and efficiency detection in the detection of VOC markers. 

### 2.2. Data Processing

The data processing pipeline in our research is comprised of three stages: A. Disassembly of the raw data into peak lists using the MS-DIAL software tool. B. Reading peak lists and merging them into a unified list per group. C. Remove blank compounds and compare pairs of groups to isolate distinguishing compounds. The unsupervised execution of stages B and C relies on the development of an algorithm that correctly pairs compounds (as EI-MS spectra) at various concentrations. A common strategy to align a compound in a complex mixture with another in other samples is by using a unique identifier, such as its RT and base peak *m*/*z* value. This simple combination, as well as alignment by library search results, may be insufficient for an alignment algorithm for GC-HRMS of small volatile compounds due to limitations in the spectral level that will be discussed herein.

**Figure 1:**
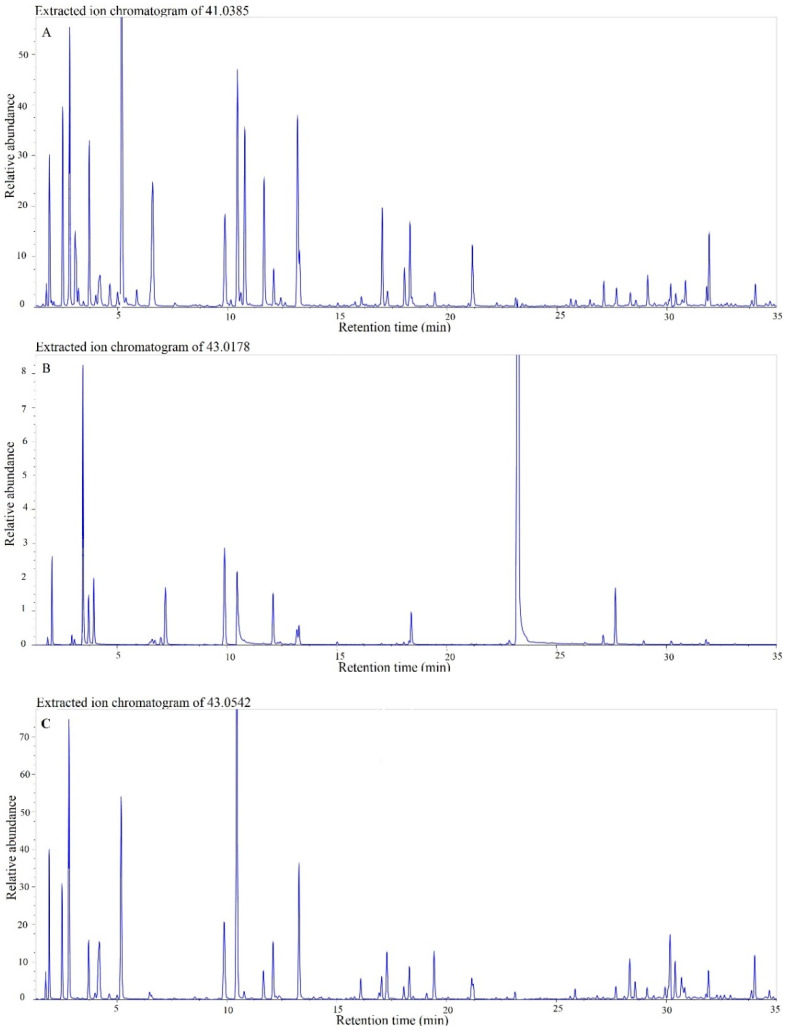
Extracted Ion Chromatogram (XIC) of a 1 nL/L sample. (**A**) XIC of *m*/*z* 41.0385; (**B**) XIC of *m*/*z* 43.0178; (**C**) XIC of *m*/*z* 43.0542.

Volatile metabolites in exhaled air are mainly composed of several atoms of carbon and hydrogen with modest-to-sparse content of heteroatoms—oxygen, nitrogen, and sulfur [32]. The low variability in molecular composition results in a high degree of similarity between the ions in the spectra of the analytes, with a high density of base peak ions around the mass range of *m*/*z* 40–45. The compounds we selected to represent breath VOCs significantly differ in their molecular formula, yet 12 of these compounds have a base peak in the range of *m*/*z* 41–45. The high mass resolution and mass accuracy of GC-Orbitrap is of great value by separating isobars, such as *m*/*z* 41.026 (C_2_H_3_N^+^) and *m*/*z* 41.038 (C_3_H_5_^+^), *m*/*z* 42.034 (C_2_H_4_N^+^) and *m*/*z* 42.046 (C_3_H_6_^+^), and *m*/*z* 43.018 (C_2_H_3_O^+^) and *m*/*z* 43.054 (C_3_H_7_^+^). The high density of three of these ions is presented in Figure 1, including the isobars *m*/*z* 43.018 and *m*/*z* 43.054 taken as XIC from a sample at a concentration of 1 nL/L. Alignment of the ions as features, RT or RI coupled with accurate mass *m*/*z*, will bring ambiguity when taking into consideration chromatographic drifts. In many cases, these ions are not only present but also prominent or dominant. For example, around RT = 4 min, a compound with a base peak at 41.026 elutes 0.12 min apart from another that has a base peak at 41.038, with methyl acetate in between with a base peak at 43.018. Similarly, around 10.4 min, a compound with a base peak at 43.018 elutes only 0.03 min before another that has a base peak at 41.038 and a second highest (>90%) ion at *m*/*z* 43.054. On the other hand, the use of accurate mass MS requires consideration of the fluctuation in measured mass, so the measured mass of a base peak of the same compound might slightly change from one sample to another. 

Moreover, among the selected model compounds, we can find pairs of compounds that largely differ in their mass and structure but still have identical base peaks. For example, both *n*-Pentane and 3,3-Dimethyl-2-Butanone share a base peak of *m*/*z* 41.038, as is the case with 2-Pentanone and Ethyl acetate; both have a base peak at *m*/*z* 43.018. While we selected the markers to be easily separated in time with the GC, in real-world samples, pairs of almost co-eluting compounds, which are closer in their composition and structure, will result in a high probability of identical base peaks. The uneven base peak density of breath volatiles, with emphasis on low mass, implies that the simple combination of RT and base peak *m*/*z* as a tag for the alignment of compounds between samples is not necessarily unequivocal, even when using an accurate mass. For example, around 5.2 min, there are two compounds with a base peak of 41.038 and ΔRT < 0.2 min, a short interval that may cause confusion if an alignment solely based on the base peak is employed. However, the combination of high resolution in chromatography and MS enables one to use RT and base peak with margins of ΔRT < 0.12 min and Δmass < 1.1 mDa as convenient tags for the initial alignment of compounds between samples. 

**Figure 2:**
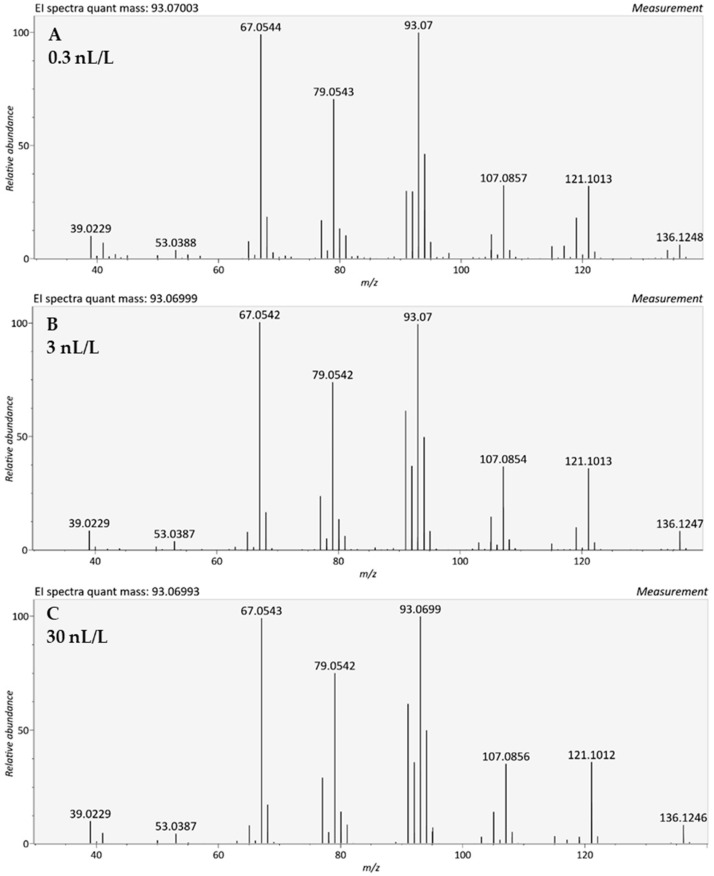
Limonene spectra using TD-GC-MS (Orbitrap) at three different concentrations. (**A**) 0.3 nL/L, where the dominant ion is *m*/*z* 93.070; (**B**) 3 nL/L, where the dominant ion is *m*/*z* 67.0541; (**C**) 30 nL/L, where the dominant ion is *m*/*z* 93.070.

**Figure 3:**
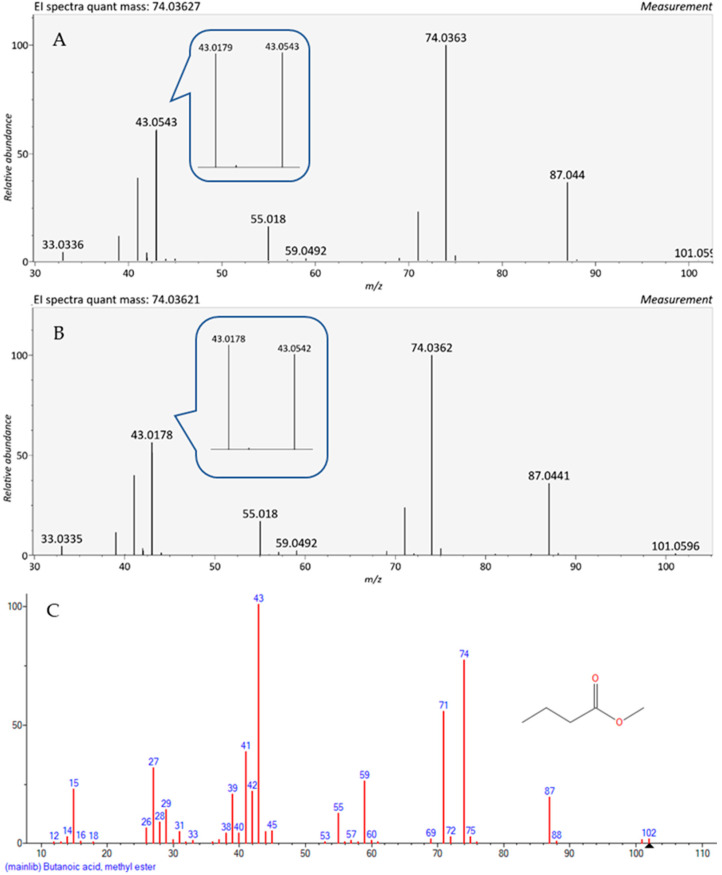
Methyl butyrate spectra using TD-GC-MS (Orbtriap) at two different concentrations: 0.3 nL/L and 1 nL/L. (**A**) At 0.3 nL/L the dominant ion has *m*/*z* 74.036 and the second ion has *m*/*z* 43.054; (**B**) At 1 nL/L the dominant ion has *m*/*z* 74.036, while the second ion has *m*/*z* 43.018; (**C**) NIST library for the same compound, where the dominant ion is 43 and the second is 74.

However, as we try to push the limit of detection toward a few nL/L and below, statistical noise and background interferences induce small fluctuations in extracted spectra. While examining the results of low-nL/L samples, we encounter cases in which we lose the anchor of the base peak. In spectra with a predominant second ion, sometimes the first and second ions are swapped, and the latter is extracted as the base peak. This is the case with limonene, as demonstrated in Figure 2. Its spectrum is dominated by ions at *m*/*z* 93.07 and *m*/*z* 67.054, but their order of intensities is sometimes swapped at concentrations of 10 nL/L and below. Thus, at 3 nL/L, the second abundant ion at *m*/*z* 67.054 becomes a base peak instead of the *m*/*z* 93.07 ion. Similarly, the first (59.049) and second (73.065) ions of 2-methyl-2-butanol swap order in some spectra at 1 nL/L and below. The addition of the second abundant ion as an additional anchor for peak alignment is effective for most compounds, but even then, we have encountered cases in which the second and third ions swap their order of intensity. In Figure 3, we present the spectra of methyl butyrate, taken at concentrations of 0.3 and 1 nL/L. The two top spectra are very similar, but small fluctuations in the intensities result in a swap between the second and the third ions at the lower concentration. These deviations in relative abundance of spectral ions are minor, few present in relative abundance, and will not mislead an experienced mass-spectrometrist or interfere with library search. However, it might induce discrepancies in an algorithm-based alignment since the order of ions in the spectrum is inconsistent.

In general, as demonstrated in both Figure 2 and Figure 3, the structure of the spectra is stable along the concentration range due to the high performance of mass spectrometry and peak deconvolution. A comparison between the HRMS spectra and NIST library low concentration spectrum shows similarities, but also several differences (Figure 3); the high mass resolution of the Orbitrap splits the *m*/*z* 43 ion in the library to *m*/*z* 43.018 and *m*/*z* 43.054, so that *m*/*z* 74 becomes the dominant peak. Moreover, the spectrum of methyl butyrate shows additional deviations of ion intensity in the experimental spectrum when compared with the NIST library. The ions at *m*/*z* 59 and, to a lesser extent, *m*/*z* 71, are substantially lower in the experimental spectrum of GC-Orbitrap in a way that can decrease the score of library match. Similarly, in the NIST library spectrum of limonene, we can see a dominant ion at *m*/*z* 68.062, which suffers a significant decrease in Orbitrap GC-MS.

### 2.3. pyAIR—In-House Algorithm

**Figure 4:**
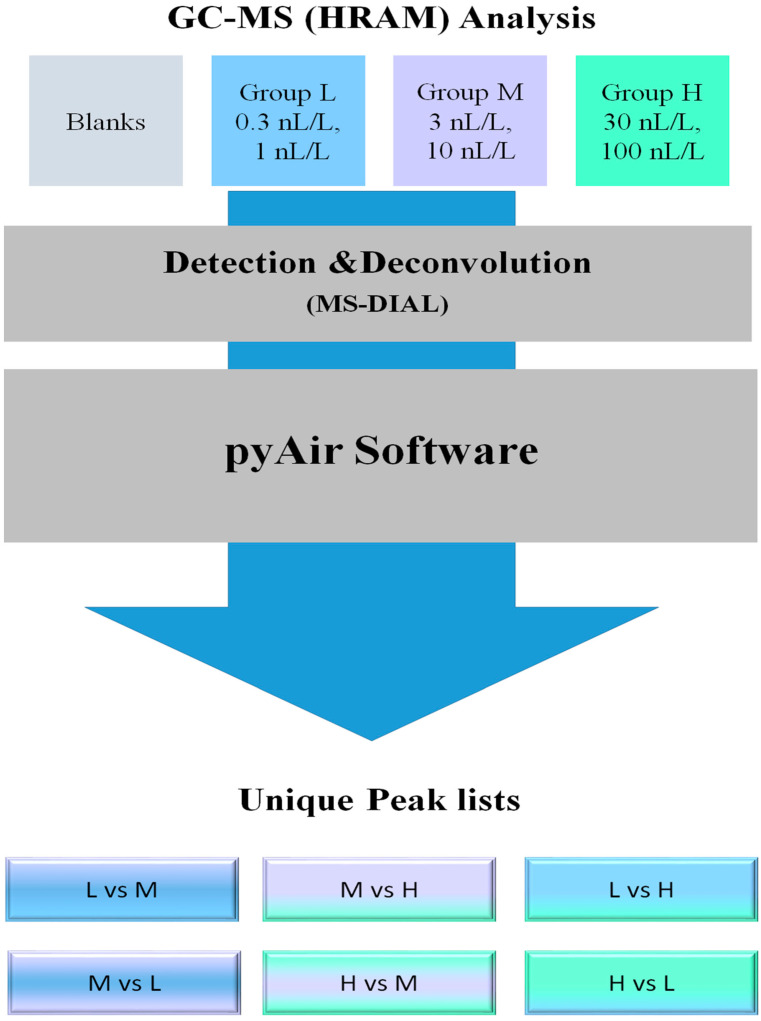
Data processing pipeline for finding biomarkers in breath analysis. The first step is analysis using GC-MS-HRMS. The second step is detection and deconvolution of the peaks using MS-DIAL. The third step is using pyAir to find unique peaks for each group.

In general, pyAIR was designed as a software tool that accepts peak lists of all samples and, in an unsupervised operation, performs an untargeted search for compounds that distinguish each group versus the others, as shown in Figure 4. 

Three software packages were used as interfaces between the raw data coming from the GC-MS (Orbitrap) and pyAIR. Using ProteoWizard MS-Convert [33], MS data files are converted from *.raw to *.mzML, and then further converted to *.abf format using ABF-Converter [34]. MS-DIAL [28] reads the *.abf files to perform peak detection and deconvolution for each file. The MS-DIAL results are exported as peak lists in separate files, using a *.msp format that can be read as text. 

**Figure 5:**
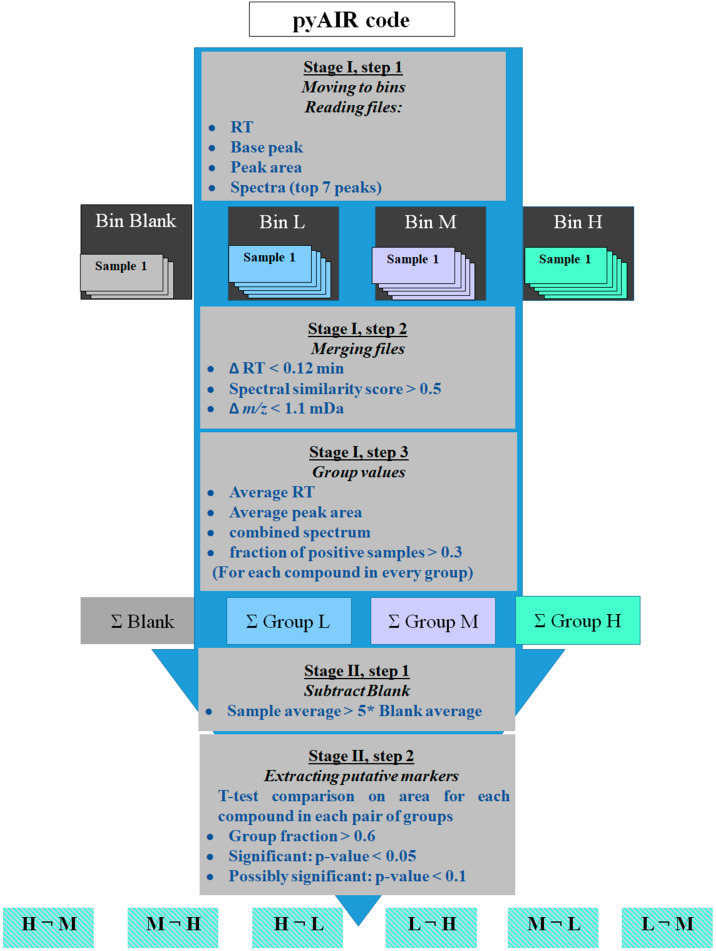
pyAIR algorithm description; after peak detection and deconvolution by MS-DIAL, pyAIR is moving to bins, grouping, performing blank subtraction, groups comparison, and statistics until finding putative biomarkers. The algorithm is suitable for a large number of groups that can be individually processed, and then compared to each other.

pyAIR processes the data in two stages: stage I merges the samples into groups, while in stage II, the groups are compared to find the distinguishing compounds. In stage I, as described in Figure 5, pyAIR starts by reading a list of file names and their classification into groups. Following the list of files, pyAIR reads the data from the *.msp files, and for each compound, a record is created to store spectral details: RT, base peak, chromatographic peak area, and partial spectrum (top seven ions) normalized to the base peak. All records are accumulated in a dataframe per data file, and these dataframes are sorted into bins according to the sample classification in the list file. 

The next step is to merge the peak lists, one bin after another, into a unified comprehensive list of compounds for each group of samples. The merging of records is based on RT, and accurate mass spectral similarity. The accuracy limits for RT and the mass of ions were set based on our experience with the robustness of RT and mass measurement achieved according to instrument maintenance and the experimental method used. The prerequisite for the alignment of the two compounds from the different samples was set as: ΔRT < 0.12 min and spectral similarity score > 0.5 with Δ *m*/*z* < 1.1 mDa. The spectral similarity score is in the range of 0–1, where a higher score represents a better spectrum similarity. To avert the effects of intensity difference on similarity score and success alignment, the spectral similarity is calculated as a dot product of the normalized spectra vectors:

(1) $$\mathrm{Score}=0.5\;\times \;\frac{U\times V}{\sqrt{U\times U}\times \sqrt{V\times V}}+0.25\;\times \;\frac{U`\times V`}{\sqrt{U`\times U`}\times \sqrt{V`\times V}`}+0.25\;\times \;\frac{U``\times V``}{\sqrt{U``\times U``}\times \sqrt{V``\times V``}}$$

- U and V are the original spectra (top 7 ions).
- U` and V` are the spectra without the base peak of spectrum U.
- U`` and V`` are the spectra without the base peak of spectrum V.

Using the dot product for similarity evaluation, this equation weighs similarity equally with and without the base peak. The firs term is a simple dot product of two spectra, which is a common way to calculate spectral similarity [35]. However, when the base peak is much higher than the other ions, the similarity of ions 2–7 has a marginal influence on the score, so the second and third terms were added to the expression to better examine the similarity of less abundant ions. In terms II and III, the base peaks of spectra U and V (respectively) are separately omitted from the calculation of the dot product since the base peaks are not necessarily identical. Weighing the three terms gives a balanced score of the spectral similarity. pyAIR stores and uses only the top seven ions (by intensity) of each spectrum since the contribution of the rest is marginal to the similarity score.

To set the ground for statistical comparison between groups, in the last step of stage I the content is tallied for each record in each group: non-zero peak areas are kept in a list, RTs are averaged, and all the spectra are combined to a single spectrum. Compounds that appear in less than 30% of samples are removed from the list (keeping compounds with a fraction > 0.3).

Stage II of pyAIR compares groups, searching for compounds that are unique to one group versus the other. Stage II is comprised of two steps, the first of which is the comparison of each sample group to the blank group. Applying the uniqueness condition of the average peak area of sample > 5× Average peak area of blank eliminates contaminant compounds that originate from the air bag, TD adsorbent, or the GC system. The second step is inter-comparison of the sample groups based on T-test calculation for each compound to assess its potential as a marker. The classification of a marker was as follows: a stricter condition of fraction > 0.6 (present in >60% of samples) was applied to every compound in the list, to ensure a high robustness of the marker; a marker is considered “significant” when *p*-value ≤ 0.05 and “possibly significant” when 0.05 < *p*-value < 0.1. The final step is the export of the marker lists for each group to Excel for further examination, as described in Figure 5.

### 2.4. Detection of Group Markers 

For data processing of the experimental results, MS-DIAL was limited to the RT range of 1–35 min and the MS range of 30–300 Da, ranges that safely contain all VOCs, even phenol, which is the least volatile VOC in the mixture used. Peak detection and deconvolution of raw data yielded peak lists of 200–300 compounds per sample, a number that reflects the natural background of the TD-GC-MS system. Merging of four sample peak lists for each sample group yielded unified peak lists that contain 300–400 compounds per group. Examination of the peak lists shows that all compounds were detected in all samples, except for two, propanol and 2-methoxy-ethanol, which were not detected in samples with the lowest concentration of 0.3 nL/L. 

The three sample groups were compared to the blank group using spectral alignment and uniqueness conditions, as mentioned above. After the elimination of blank compounds and imposing the condition of a fraction >0.3, we were left with three reduced peak lists, one for each group, with 30–60 compounds per group. All model compounds were present in the merged lists with fraction = 1, except for propanol and 2-methoxy-ethanol in group L, which had fraction = 0.5. Due to the high phenol levels in the blank, it was removed from all lists. Pentane was removed from the L list due to its level in the blank. The following step was inter-comparison of the L, M, and H groups in both directions, using spectral alignment of the compounds, *p*-value, and a fraction > 60%. 

**Figure 6:**
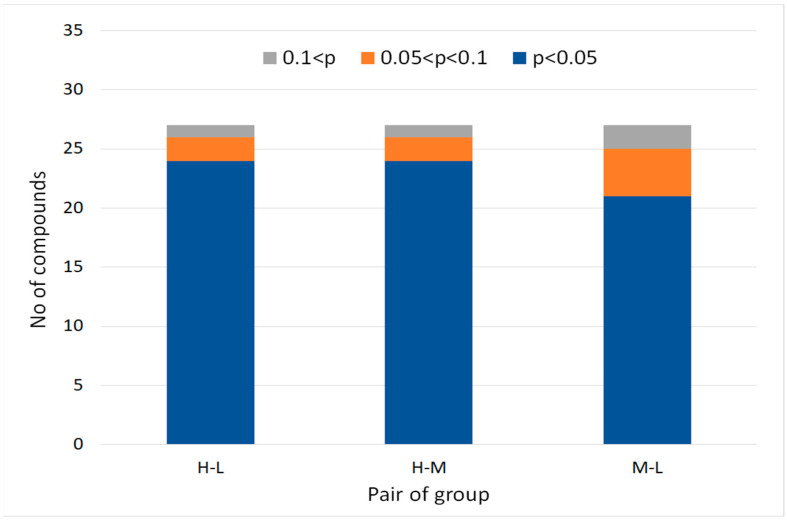
Number of model compounds found in each group. Group H represents the higher concentrations, 30 nL/L and 100 nL/L in 4 different samples. Group M represents the medium concentrations, 3 nL/L and 10 nL/L in 4 different samples. Group L represents the low concentrations: 0.3 nL/L and 1 nL/L. Three different comparisons are shown: H-L, H-M, and M-L. The blue color represents *p*-value lower than 0.05, the orange represents *p*-value higher than 0.05 and lower than 0.1, and the gray color represents disqualified compound with *p*-value higher than 0.1.

A graphical representation of the workflow results is presented in Figure 6. Comparison between the H and the L groups reveals that 24 compounds were found to be significant (*p*-value < 0.05), while phenol was correctly removed as aforementioned. 6-Methyl 5-hepten 2-one and Limonene were found at lower significance (0.05 < *p*-value < 0.1) due to the high standard deviation in group H. Comparison between groups H and M yielded similar results. Comparison between groups M and L shows that 21 compounds were found to be significant, even at concentrations in the low nL/L range. Four compounds were found to be possibly significant; three had an average M > 5*Average L and *p*-value < 0.075 but also a high STDEV in the L group. One compound, pentane, had a high background level; thus, it was disqualified. Other hydrocarbons, such as the alkanes and aromatic compounds, which are also known to be excreted from polymers, were detected in the blank but at concentrations low enough not to be excluded from the sample groups. The lists of significant compounds contained, at most, 10 compounds in excess, compounds that were not spiked to the stock bag.

## 3. Discussion

pyAIR was developed to complete a multistage TD-GC-HRMS workflow for the successful handling of breathomics challenges with fit-to-purpose sampling, analysis, and data processing tools. TD tubes are known to be beneficial for sampling VOCs from air in terms of sensitivity, accuracy, and precision [36]. These tubes are compatible with Owlstone Medical’s breath biopsy instrument, the ReCIVA breath sampler device, which is easy to use and can distinguish between end-tidal and other breathing phases [37]. While GC separates the compounds along the time axis, EI/HRMS ionizes and fragments the compounds to reveal structural information and separates the ions in the *m*/*z* axis, including separation of isobars [38], such as *m*/*z* 43.018 (C_2_H_3_O^+^) and *m*/*z* 43.054 (C_3_H_7_^+^). This high power of separation is in need in view of the elemental composition similarity of breath metabolites [20]. The analysis output is a large data set of EI/MS single stage ionization and fragmentation, with partial separation of the compounds. A complementary step of deconvolution of the total ion count (TIC) to compounds is required before the differences between samples can be studied [39]. MS-DIAL is a well-established, accessible, and supported tool for this purpose [28], and the study results show successful performance below 1 nL/L in the presence of abundant matrix compounds from the Tenax absorbent.

Multi-group data processing is required to detect putative biomarkers and distinguish between different groups of illnesses, such as “sick” and “healthy” or stages of illness. The search for compounds that differentiate between sample groups begins with the alignment of compounds in all samples so that each compound can serve as an axis on which the samples are positioned according to peak area that reflects its concentration in the sample. The alignment step is essential, regardless of the computational method used later—statistics on each compound separately or PCA for all compounds together. Correct alignment is crucial for the next steps of data processing because alignment of unrelated compounds or failure to align the same compound in different samples will disrupt the statistical evaluation step. While in tandem-MS results, the combination of RT and accurate mass precursor ion is considered unequivocal for alignment, with EI/MS the fluctuation of intensities in the spectrum may cause misalignment due to inconsistency in the order of ions. As demonstrated above, the base peak might change for compounds with a strong second ion (such as limonene and 2-methyl-2-butanol), while a supporting second ion might change when a third ion is close in intensity (such as methyl butyrate). Moreover, an additional difficulty arises from the uneven distribution of *m*/*z* of base peaks between *m*/*z* 41–45, as demonstrated with our model compounds (12 out of 27), with high occurrence of *m*/*z* 41.038, 43.018, and 43.054, for example. To overcome these challenges, we adopted the combination of RT with spectral similarity as an unequivocal tag for alignment. The calculation of spectral similarity is based on the dot-product method [40], which is carried out twice, with and without the base peak (1:1 weight), to compensate for compounds with a weak second ion. Spectral similarity was calculated for the top 7 ions, considering the contribution of the remaining ions to be negligible. The separation of samples into several groups is important to achieve a trustworthy reflection of multi-variate diagnoses, such as illness stage, symptomatic state, gender, etc. As mentioned, the importance of spectral similarity-based alignment stems from the high similarity of fragments between different VOCs and fluctuation in ions’ relative intensity that alters the order of ions, potentially leading to errors if only a base peak or two-peak alignments are used. On the other hand, the use of MS library identification as an alignment tag cannot be trusted, since search results for small VOCs at low concentrations are severely affected by the fluctuations in ion intensities.

To test the performance of pyAIR, a set of samples was prepared with 27 model volatiles spiked to Tedlar bags filled with nitrogen. To simulate breath metabolites, all the model compounds are present in the breath of healthy people [41]. According to the literature, low concentrations are expected [42]; hence, the concentrations of the model compounds were in the range of 0.3–100 nL/L. The gaseous samples were sampled onto TD tubes using GC-HRMS for analysis followed by MS-DIAL processing for peak detection and deconvolution. pyAIR was used to align the compounds between samples, merge them separately in each group, and compare peak intensities between the groups in order to detect markers.

To simulate the abundance variability in the groups and between them, we decided to divide the spiked samples into three groups: Low: 0.3 nL/L and 1 nL/L, Medium: 3 nL/L and 10 nL/L, and High: 30 nL/L and 100 nL/L. The difference in concentration of the group markers simulates discrimination between stages of illness based on concentration, which can be in the realistic range for real-case scenarios [43].

All the detected compounds were correctly aligned to create a unified list for each group, using orthogonal conditions on RT (ΔRT < 0.11 min) and spectral similarity (score > 0.5) to validate the compound alignment. To handle future drifts in background level, a strict condition of Average area (Sample) > 5× Average area (Blank) is imposed on compounds after alignment of sample groups with blank group, and background compounds were removed. The final step is a comparison between each pair of sample groups, with alignment of the compounds using a T-test to determine for each compound whether it is significantly different between the groups. The comparison between groups is not symmetrical–when comparing one sample group to another, a reference group, the first should have a fraction > 0.6, while the latter should have a fraction > 0.3. The unsupervised operation of pyAIR on the data set yielded six lists of differentiating compounds, two for each pair (both directions). Each list contains two levels: significant (*p*-value < 0.05) and possibly significant (0.05 < *p*-value < 0.1). While the use of a T-test with a limit set to *p*-value < 0.05 is common, the raw data values of intensity for each compound are accessible to the analyst. Thus, the use of a different *p*-value or other statistical tools that might be more appropriate for a specific design of experiment can be easily applied.

Examination of the lower concentration groups, M vs L, shows that pentane and phenol were correctly disqualified (high background level), 21 compounds were classified as significant, and the remaining four were classified as possibly significant. Examination of the H vs. L and M groups naturally gave better results–only phenol was disqualified, while 24 and 23 compounds (respectively) were found significant. While 200–300 compounds were found in each sample, in the six comparisons of groups, up to 10 non-spiked compounds were found to have a *p*-value < 0.05, a very low percentage of false positive results. These results demonstrate the suitability of pyAIR for unsupervised data processing of multi-group TD-GC-HRMS in the field of breathomics. 

## 4. Materials and Methods

### 4.1. Chemicals and Air Bags

Five-liter Tedlar bags were purchased from Restek (number 22052). *n*-Pentane, heptane, benzene, toluene, *o*-xylene, isoprene, limonene, *tert*-butyl methylether, butylethyl ether, 2-pentanone, 3,3-dimethyl-2-butanone, 6-methyl-5-heptane-2-one, isopropyl acetate, methyl propionate, methyl butyrate, propanol, *tert*-butanol, 2-methyl-2-butanol, 3-hexanol, 2-methyl-3-pentanol, 5-methyl-3-hexanol, 2-methoxy ethanol, and phenol standards were purchased from Sigma Aldrich Israel (Rehovot, Israel). Hexane and butanol standards were purchased from Merck (Merck Millipore, Darmstadt, Germany), and methyl acetate and ethyl acetate standards were purchased from J.T. Baker (Phillipsburg, NJ, USA).

### 4.2. TD Tubes

Biomonitoring, inert-coated thermal desorption (TD) tubes, C2-CAXX-5149, were purchased from Markes International (Llantrisant, UK) and conditioned using a TC-20 conditioner at 320–330 °C for 1.5 h. Model compounds were pumped from the Tedlar bags to the TD tubes for 1 min at 0.3 L/min (0.3 liter sampling volume) [44]. Considering the volume of tidal breath, we chose to work with sample volumes of 0.3 L. 

### 4.3. GC-MS Instrumentation and Methods

Instrument: Q Exactive GC Orbitrap GC-MS/MS (Thermo Fisher, Waltham, MA, USA) coupled with a TD100-XR Thermal Desorption unit (Markes). Column Description: TSP26059-3320 TG-624SILMS GC Column, 30 m × 0.25 mm × 1.4 µm, Thermo.

Method Parameters: TD tubes were desorbed at 280 °C for 5 min and focused on a Material Emissions cold trap (Markes) held at 20 °C (trap low) at a flow rate of 50 mL/min during tube desorption. Trap desorption was carried out over 5 min at 290 °C with a 5:1 split. 

The desorbed compounds were separated on a Thermo TG-624SILMS capillary column with a 1.5 mL/min constant flow of helium carrier gas (99.9999%), utilizing the following program: 35 °C for 7 min, followed by an increase to a final temperature of 250 °C at a rate of 4 °C/min. The final temperature was held for 19 min. Q-Exactive was operated in MS mode, and electron ionization (EI) mode at 70 eV was used with a 60,000 resolution over a mass range of *m*/*z* 33–490. The transfer line and ion source temperatures were set to 230 °C.

### 4.4. Samples

Stock bag and gas samples (Table 1) were prepared as follows:

Stock bag—For each compound, to prepare a 200 μL/L stock sample, a pure material was injected as liquid into a 5 L Tedlar bag filled with 1 L of pure nitrogen. The volume to be injected into the bag was calculated according to the equation:

(2) $$V_{\mathrm{liq}}\;(\mathrm{mL})=\frac{P\;\times \;\mathrm{Vgas}}{R\;\times \;T}\times \frac{M}{d}\;=8.2\;\times \;10^{-6}\;\times \;\frac{M}{d}$$

P—pressure = 1 atm
Vgas—volume = 0.2 mL
R—specific gas constant = 8.2 × 10^−2^ L atm/(K mol)
T—temperature = 298 K
M—molar mass (g/mol)
d—density (g/mL)

As it is not feasible to spike the calculated volumes (less than 1 µL) directly into the bag, from each compound 20 times, this volume was mixed in a vial (for all 27 compounds), and 5% of the mixture was spiked into the stock bag. This Tedlar bag was left for 24 h in the fume hood at room temperature in order for the compounds to fully evaporate in the bag.

Gas samples—All gaseous samples were prepared in a 5 L Tedlar bag filled with 1 L of pure nitrogen. The stock bag at 200 μL/L was diluted to bags at 1 μL/L and 3 nL/L, and from these bags, the rest of the samples were prepared by dilution to obtain the following concentrations (*v*/*v*); 300 pL/L, 1 nL/L, 3 nL/L, 10 nL/L, 30 nL/L, and 100 nL/L.

### 4.5. Data Processing

MSConvert as part of ProteoWizard [33] ver 3.0.21349 was used for format conversion of raw files to mzML format, which was converted to *.abf format using AbfConverter 1.3.7815 [34]. 

MS-DIAL [45] ver 4.70 by RIKEN was used for peak detection and peak deconvolution, and the output was exported as peak lists in *.msp format. The main operational parameters were set as follows: data type accurate profile, RT window 1–40 min, *m*/*z* window 30–300 Da, mass accuracy 0.005, mass slice width 0.05, and Sigma window value 0.05.

pyAIR software tool was written in Python 3.9.7 (64 bit) using a Spyder IDE 5.1.5 environment that was installed as part of the Anaconda 3 [46] 2021.11 package.

## 5. Conclusions

Breathomics, a comprehensive analysis of volatile metabolites in exhaled air, is drawing much interest due to its noninvasive sampling. The unique challenges of compound alignment and comparison in the analysis of VOCs by GC-MS are better resolved with HRMS analysis, combined with pyAiR processing. pyAIR completes a workflow for unsupervised detection of VOC markers in GC-MS analysis results in a multi-group set of air samples. Tested on an evaluation set of samples, pyAIR correctly aligned all model compounds and removed background impurities. More than 80% were found to be significant (*p*-value < 0.05) down to sub nL/L range, with a very small number of false positives. The operation of pyAIR relies on compatibility with MS-DIAL and can also be easily coupled to AMDIS, both cross-platform tools for GC-MS peak detection and deconvolution, rendering it a wide range of applicability, extending the scope of TD-GC-HRMS for breathomic applications.

## Figures and Tables

**Table 1:** Model analytes with their physical properties and GC retention time (RT).

Name	Density (g/mL 25 °C)	Molar Mass (Da)	B.P. (°C)	RT (min)
*n*-Pentane	0.63	72	36	2.5
*tert*-Butanol	0.77	74	82	2.8
Isoprene	0.68	68	34	3.1
MTBE (*tert*-Butyl methyl ether)	0.74	88	55	3.1
Methyl acetate	0.93	74	57	3.9
Hexane	0.66	86	69	5.2
Propanol	0.80	60	97	5.9
Butylethyl ether	0.75	102	92	6.6
Ethyl acetate	0.9	88	77	7.2
Mehtyl propionate	0.92	88	80	8.1
Benzene	0.87	78	80	9.3
2-Methoxyethanol	0.97	76	124	9.5
Isopropyl acetate	0.87	102	89	9.9
2-Methyl-2-butanol	0.81	88	102	9.9
Heptane	0.68	100	98	10.5
Butanol	0.81	74	118	11.7
2-Pentanone	0.81	86	101	12.1
3,3-Dimethyl-2-Butanone	0.8	100	106	13.2
Methyl butyrate	0.9	102	102	13.3
Toluene	0.86	92	111	15.3
2-Methyl-3-pentanol	0.82	102	127	17.0
3-Hexanol	0.82	102	135	17.7
5-Methyl-3-hexanol	0.83	116	153	21.1
*o*-xylene	0.88	106	144	21.9
6-Methyl-5-heptane-2-one	0.85	126	173	27.1
Limonene	0.84	136	176	27.6
Phenol	1.07	94	182	30.7

## Data Availability

Data is contained within the article.

## Sample Availability

Samples of the compounds are not available from the authors.
